# Increased post myocardial infarction complications in COVID-19 outbreak: Is it dilemma?

**DOI:** 10.34172/jcvtr.2020.56

**Published:** 2020-11-24

**Authors:** Azin Alizadehasl, Bahram Mohebbi, Shirin Habibi Khorasani

**Affiliations:** ^1^Cardio-Oncology Research Center, Rajaie Cardiovascular Medical & Research Center, Tehran, Iran; ^2^Cardiovascular Intervention Research Center, Cardio-Oncology Research Center, Rajaie Cardiovascular Medical & Research Center, Iran University of Medical Sciences, Tehran, Iran; ^3^Echocardiography Department, Rajaie Cardiovascular Medical & Research Center, Tehran, Iran

**Keywords:** Myocardial Infarction, COVID-19, Complications, STEMI

In December 2019, the novel coronavirus disease 2019 (COVID-19) outbreak appeared with severe acute respiratory distress syndrome in Wuhan, china. The disease soon began to spread all over the world; so that, it was literally announced by World Health Organization (WHO) as a pandemic in March 2020.^1^ Since then, the disease has affected millions of people and became a huge matter of concern for the world health policies.

Although not yet fully understood, several cardiovascular manifestations of COVID-19 have been described. It can be presented with acute myocardial infarction (MI), myocarditis, cardiomyopathies (including stress cardiomyopathy), coronary spasms and etc. Besides, the occurrence of COVID-19 in patients with cardiac disease may be associated with higher risks and aggravate their prognosis.^2^

With the wide and quick spread of disease, the health care policies have shifted from elective procedures to management of infected patients and control of further disease growth. This issue has interfered with the usual health care of patients including the usual cardiovascular care that have been provided in recent decades.^2^ Acute ST-segment-elevation myocardial infarction (STEMI) is one of main cardiovascular emergencies with well-known mortality and morbidities, which have been significantly improved in outcome with timely management and tremendous advancements in coronary interventions.^3^ There are several reports from around the world showing the significant decline in early management of STEMI patients since COVID-19 pandemic. They have mentioned that the fear of people and strict “stay home” recommendations may have prohibited the early call for medical help; furthermore, the overwhelming of health care system with the COVID-19 patients may have caused missing of STEMI patients or significant delays in their diagnosis and management.^2,4^

It is anticipated with the aforementioned evolving issues in the management of STEMI patients, early and late complications of acute STEMI -which had been significantly reduced in the past decades- would remarkably increase. In COVID-19 era, there seems to be an increase in mechanical and electrical complications of STEMI. There have been reports of post-STEMI ventricular septal rupture, cardiogenic shock, tamponade, Cardiac rupture and severe MR.^5,6^ We assume that the following reasons may be responsible: (1) late hospital arrival due to fear of infection by the patient; (2) late diagnosis due to misinterpretation of chest discomfort with COVID-19 presentations and subsequent time delay in proper intervention; (3) increasing door-to-balloon time with the need of providing protective equipment for health-care workers.

We recommend that there should be an organized guideline in the management of STEMI patients during COVID-19 era; so that, the burden of late cardiovascular complications remaining after the COVID-19 pandemic would be less. We also think there is a need to inform cardiac patients not to ignore their symptoms and provide online gadgets for communications with eligible people to reduce the risk of missing acute cardiac events during this outbreak.
